# Consensus statements for pharmacological management, monitoring of therapies, and comorbidity management of psoriatic arthritis in the United Arab Emirates

**DOI:** 10.1111/1756-185X.14406

**Published:** 2022-08-02

**Authors:** Khalid A. Alnaqbi, Suad Hannawi, Rajaie Namas, Waleed Alshehhi, Humeira Badsha, Jamal Al‐Saleh

**Affiliations:** ^1^ Department of Rheumatology Tawam Hospital Al Ain UAE; ^2^ College of Medicine and Health Sciences UAE University Al Ain UAE; ^3^ Emirates Health Services (EHS) Dubai UAE; ^4^ Ministry of Health and Prevention Dubai UAE; ^5^ Division of Rheumatology, Department of Internal Medicine Cleveland Clinic Abu Dhabi UAE; ^6^ Medcare Hospital Dubai UAE; ^7^ Dr. Humeira Badsha Medical Center Dubai UAE; ^8^ Dubai Hospital Dubai UAE

**Keywords:** biologics, comorbidities, disease‐modifying drugs, domains, dosage, monitoring, pharmacological management, psoriatic arthritis, recommendations

## Abstract

**Objective:**

Psoriatic arthritis (PsA), a chronic inflammatory disease characterized by heterogeneous clinical manifestations, substantially impacts the quality of life of affected individuals. This article aims at developing consensus recommendations for the management of PsA and associated comorbidities and screening and monitoring requirements of PsA therapies in the United Arab Emirates (UAE) population.

**Methods:**

An extensive review of present international and regional guidelines and publications on the pharmacological management, monitoring of therapies in the context of PsA was performed. Key findings from guidelines and literature were reviewed by a panel of experts from the UAE at several meetings to align with current clinical practices. Consensus statements were formulated based on collective agreement of the experts and members of Emirates Society for Rheumatology.

**Results:**

The consensus recommendations were developed to aid practitioners in clinical decision‐making with respect to dosage recommendations for pharmacological therapies for PsA, including conventional drugs, non‐biologic, and biologic therapies. Consensus recommendations for therapeutic options for the treatment of PsA domains, including peripheral arthritis, axial disease, enthesitis, dactylitis, psoriasis, and nail disease, were developed. The panel emphasized the importance of monitoring PsA therapies and arrived at a consensus on monitoring requirements for PsA therapies. The expert panel proposed recommendations for the management of common comorbidities associated with PsA.

**Conclusion:**

These consensus recommendations can guide physicians and healthcare professionals in the UAE in making proper treatment decisions, as well as efficiently managing comorbidities and monitoring therapies in patients with PsA.

## 1 INTRODUCTION

Psoriatic arthritis (PsA) is a heterogeneous chronic inflammatory disease occurring in about 30% of patients with psoriasis.^1^ The global prevalence of PsA varies by geographic region and ranges from 0.001% to 0.42%, while the prevalence of PsA is 0.01% to 0.3% in Middle East countries.^2^, ^3^, ^4^, ^5^, ^6^ Approximately 20% of patients diagnosed with PsA may develop a more aggressive form of arthritis resulting in joint damage.^4^ In patients with PsA, quality of life (QoL) is substantially impacted consequent to stress, depression, mood changes, pain, and compromised physical functioning.^7^, ^8^

Nearly all the current treatment recommendations for PsA are reflective of the treatment and disease landscape in developed countries, particularly Europe and the United States.^9^, ^10^, ^11^ Currently, not much is known about the epidemiology and treatment practices specific to PsA in the Middle East. There are several local challenges that may not be adequately accounted for, in currently available treatment recommendations for PsA.^12^

Multiple factors necessitate national recommendations for the management of PsA specific to the United Arab Emirates (UAE), including wide variability in healthcare systems, patient access to advanced care, affordability of treatment, practicing rheumatologists trained in different countries and implementing different approaches to treatment, and ethnic diversities among patients from almost 200 countries in the UAE. Many of the newer approved therapies such as biological disease‐modifying antirheumatic drugs (DMARDs) may not be accessible to patients who do not have insurance coverage, given their prohibitive cost. Other factors that preclude the implementation of global treatment recommendations in local clinical practice are lack of disease awareness among both patients and healthcare providers, shortage of healthcare resources, and lack of multidisciplinary healthcare clinics.^1^

The objectives of this article are to develop consensus statements for the pharmacological management of PsA and associated comorbidities and screening and monitoring requirements of PsA therapies, to assist practicing physicians in the UAE.

## 2 METHODS

For the development of the consensus guidelines, 6 experts with international board certifications, and more than 15 years experience in rheumatology and an interest in psoriatic arthritis representing different healthcare sectors (government and private) of the UAE were chosen from the Emirates Society for Rheumatology and convened in several meetings.

A targeted literature review was conducted. Current international and local treatment guidelines for PsA were identified through an extensive literature search and reviewed by members of the panel to identify unmet needs in local treatment practices in the UAE. Regional guidelines were compared with the latest international guidelines from the American College of Rheumatology/National Psoriasis Foundation (ACR/NPF) Guideline for the Treatment of Psoriatic Arthritis 2018, European League Against Rheumatism (EULAR) 2019, The Group for Research and Assessment of Psoriasis and Psoriatic Arthritis (GRAPPA) 2020 Update, GRAPPA 2015 (detailed), and 2014 Saudi Practical Guidelines on Biologic Treatment of Psoriasis.^9^, ^10^, ^11^, ^13^, ^14^

As of August 2021, there is a dearth for guidelines on management of PsA specific to the Arab region.

Based on a review of international and regional guidelines, consensus statements were developed focusing on pharmacological treatment options, screening and monitoring requirements for PsA therapies, and management of comorbidities associated with PsA. The results were discussed with all the members of Emirates Society of Rheumatology meeting to arrive at the final consensus statements. Key findings from the review were presented as statements.

The key objectives of the meetings were:

1. to critically review the available regional and international recommendations on the management of patients with PsA
2. to develop regional recommendations for effective management of PsA
3. to develop regional recommendations for screening and monitoring requirements for PsA therapies.

Several meetings were held to generate consensus statements regarding pharmacological management of psoriatic arthritis. The first expert panel meeting was conducted on September 23, 2020 and the meeting lasted for 2 hours. The second expert panel meeting was conducted on October 7, 2020 and the meeting lasted for 3 hours. The third expert panel meeting was held on December 16, 2020 and lasted for 2 hours. The fourth and fifth meetings were held in the presence of Emirates Society for Rheumatology members on December 18, 2020 and May 22, 2021 respectively; each meeting lasted for almost 2 hours. The final meeting was held on August 10, 2021 and lasted for 2 hours when the consensus statements were approved.

The consensus statements have been written in 2 separate papers. The first paper focuses on overarching principles, evaluation of PsA and non‐pharmacological treatment options of PsA.^15^ The present article, which is the second part covers consensus statements related to pharmacological management of PsA (dosing and administration recommendations, treatment recommendations for PsA domains and consensus statements on efficacy and safety profile of non‐biologic and biologic therapies), screening and monitoring requirements for therapies and management of comorbidities.

## 3 RESULTS AND DISCUSSION

### 3.1 Pharmacological treatment options

GRAPPA recommendations (2015) are centered around major subdomains within PsA, including peripheral arthritis, axial disease, enthesitis, dactylitis, psoriasis, and nail disease. In accordance with the overarching principles outlined in the GRAPPA recommendations, the expert panel agreed that treatment selection should be based on shared decision‐making between the physician and patient. The current treatment recommendations were developed to align with the core recommendations from GRAPPA.^9^

Pharmacological therapies for the management of PsA include:

- Symptomatic treatments: non‐steroidal anti‐inflammatory drugs (NSAIDs) and glucocorticoids (GCCs)^9^
- DMARDs:
  o conventional synthetic DMARDs (csDMARDs): methotrexate, sulfasalazine, leflunomide^9^
  o targeted synthetic DMARDs (tsDMARDs): phosphodiesterase‐4 inhibitor (PDE‐4i) (apremilast), Janus kinase (JAK) inhibitors (tofacitinib, upadacitinib)^9^, ^10^, ^16^
- Biologic DMARDs:
  o tumor necrosis factor inhibitors (TNFi): adalimumab, etanercept, infliximab, certolizumab pegol, golimumab^9^
  o interleukin‐12/23 inhibitors (IL‐12/23i): ustekinumab^9^
  o IL‐23 inhibitors (IL‐23i): guselkumab^17^
  o IL‐17 inhibitors (IL‐17i): secukinumab, ixekizumab^10^
  o cytotoxic T‐lymphocyte‐associated protein 4‐immunoglobulin (CTLA4‐Ig): abatacept^10^
  o upcoming therapies: filgotinib, risankizumab, brodalumab, bimekizumab, deucravacitinib^18^, ^19^, ^20^, ^21^, ^22^

### 3.2 Dosage and administration

The dosage recommendations for the pharmacological agents are shown in Table 1.

**TABLE 1:** Dosage recommendations for pharmacological therapies for PsA

Therapeutic class	Dosage	Route of administration
NSAIDs^a^		Oral and IM
GCCs^b^	Lowest effective dose	IM and IA
csDMARDs		
Methotrexate^23^	7.5–30 mg/wk	Oral, SC
Sulfasalazine^26^, ^27^	2 to 3 g/d	Oral
Leflunomide^28^	Daily loading dose of 100 mg for 3 d followed by 20 mg/d	Oral
tsDMARDs		
Apremilast^30^, ^31^, ^32^, ^33^	Apremilast 30mg twice daily (maintenance dose)	Oral
Tofacitinib^34^	5 mg twice daily (immediate release) or 11 mg once daily (extended release) in combination with non‐biologic DMARDs	Oral
Upadacitinib^16^	15 mg once daily	Oral
TNFi^b^		
Adalimumab^107^	40 mg every 2 wks in patients with PsA with inadequate response to DMARDs	SC
Etanercept^108^, ^109^, ^110^	50 mg once weekly	SC
Infliximab^111^, ^112^	5 mg/kg at 0, 2, 6 wks, and every 8 wks thereafter.	IV
Certolizumab pegol^113^, ^114^	400 mg at wks 0, 2, 4 Maintenance dose: 200 mg every 2 wks, or 400 mg every 4 wks once clinical response is confirmed	SC
Golimumab^115^	SC: 50 mg monthly IV: 2 mg/kg over 30 min at Wks 0 and 4 (loading dose), thereafter every 8 wks (maintenance)	SC, IV
IL‐12/23i^c^		
Ustekinumab^37^	45 mg or 90 mg (based on weight) at 0 and 4 wks, and every 12 wks thereafter	SC
IL‐17i		
Secukinumab^116^	300 mg in patients with concomitant moderate‐to‐ severe plaque psoriasis or who are TNFi inadequate responders at wks 0, 1, 2, 3 and 4 and every 4 wks thereafter. 150 mg in other patients at wks 0, 1, 3, 4 and every 4 wks thereafter and based on clinical response the dose can be increased to 300 mg.	SC
Ixekizumab^38^, ^117^	160 mg followed by 80 mg every 2 or 4 wks in patients who previously had inadequate response to TNFi. For patients with arthritis and moderate‐to‐severe plaque psoriasis, using the dosing regimen for plaque psoriasis;160 mg SC at wk 0, then 80 mg SC at wks 2, 4, 6, 8, 10 and 12, then 80 mg SC every 4 wks, starting wk 16.	SC
IL‐23i		
Guselkumab^40^	100 mg at wks 0 and 4, and every 8 wks thereafter (maintenance)	SC
(CTLA4‐Ig) T‐cell co‐stimulation inhibitor		
Abatacept^43^, ^118^	500 mg, 750 mg, or 1000 mg (based on weight range); following initial IV infusion, subsequent infusions should be administered at 2 and 4 wks and every 4 wks thereafter) 125 mg of abatacept injection should be administered SC once weekly	IV, SC

Abbreviations: csDMARD, conventional synthetic disease‐modifying antirheumatic drug; CTLA4‐Ig, cytotoxic T‐lymphocyte‐associated antigen4‐immunoglobulin; GCCs, glucocorticoids; IA, intra‐articular; IL‐17i, interleukin‐17 inhibitor; IL‐12/23i, interleukin‐12/23 inhibitor; IL‐23i, interleukin‐23 inhibitor; IM, intramuscular; IV, intravenous; JAK, Janus kinase; NSAIDs, non‐steroidal anti‐inflammatory drugs; PsA, psoriatic arthritis; PDE‐4i, phosphodiesterase‐4 inhibitor (apremilast); SC, subcutaneous; TNFi, tumor necrosis factor inhibitor; tsDMARD, targeted systemic disease‐modifying antirheumatic drug.

^a^ Mentioning the doses of all available NSAIDs is beyond the scope of this paper.

^b^ European League Against Rheumatism 2019 and Group for Research and Assessment of Psoriasis and Psoriatic Arthritis 2015 guidelines recommend administering the lowest effective doses of GCC.

^c^ Loading dosage might vary according to the severity of psoriasis and other domains of the disease.

#### 3.2.1 NSAIDs

The expert panel urged that a thorough safety evaluation should precede the use of NSAIDs in patients with comorbid medical conditions (eg peptic ulcer disease, chronic kidney disease [CKD], cardiovascular disease [CVD]).

#### 3.2.2 GCCs

In the treatment of peripheral arthritis, GCC is recommended conditionally, and the lowest effective doses should be administered to reduce the risk of side effects.^9^

#### 3.2.3 csDMARDs

Conventional DMARDs are indicated for the treatment of moderate‐to‐severe PsA and in patients who have failed to respond to short‐term NSAID therapy. Methotrexate has been shown to improve disease activity and health‐related QoL in patients with PsA. Methotrexate has a broad therapeutic dose range (7.5–30 mg/wk) and different administration forms (oral, or subcutaneous).^23^ Evidence suggests that monotherapy with methotrexate offers moderate improvement in joint and skin disease in patients with PsA, and doses >15 mg/wk are associated with greater clinical efficacy compared to lower doses.^24^, ^25^

In patients with mild‐to‐moderate peripheral arthritis, use of sulfasalazine at a dose of 2–3 g/d may improve functional outcomes.^26^, ^27^

Leflunomide monotherapy with a daily loading dose of 100 mg/d for 3 days, followed by 20 mg/d is effective in the management of patients with mild‐to‐moderate PsA.^28^ In an ongoing randomized, placebo‐controlled, double‐blind trial, the effectiveness of combination therapy of methotrexate and leflunomide in the treatment of patients with PsA is being evaluated, and the outcomes of the study are expected to provide key information for treatment strategies in PsA.^29^

#### 3.2.4 tsDMARDs

##### Phosphodiesterase‐4 inhibitors

Apremilast at a dose of 30 mg twice daily improves signs and symptoms and physical function in patients with active PsA.^30^, ^31^, ^32^, ^33^

##### JAK inhibitors

The recommended dosage of upadacitinib, a selective JAK inhibitor, is 15 mg once daily orally in patients with active PsA, who have not adequately responded or are intolerant to 1 or more DMARDs.^16^

According to EULAR 2019 recommendations, tofacitinib should be administered after inadequate response or intolerance to at least 1 bDMARD, or in case bDMARDs are not considered appropriate (due to patient preference for oral therapy or adherence issues to injectable formulations).^10^ The recommended dose of tofacitinib is 5 mg twice daily (immediate release) or 11 mg once daily (extended release) in combination with non‐biologic DMARDs.^34^

#### 3.2.5 Biologic DMARDs

##### TNF inhibitors

TNFi agents approved by the US Food and Drug Administration (FDA) and other health authorities worldwide for PsA treatment include etanercept, infliximab, adalimumab, golimumab, and certolizumab pegol. They are recommended for use in PsA after inadequate response to at least 1 synthetic DMARD, although they may also be used as initial therapy.^35^ TNFi agents are recommended in peripheral arthritis, and are also the first choice of therapy in enthesitis, dactylitis, and nail psoriasis.^36^

The dosage recommendations for TNFi (adalimumab, etanercept, infliximab, certolizumab pegol, and golimumab) are shown in Table 1.

##### IL‐12/23 inhibitors

The IL‐12/23 inhibitor ustekinumab has been recommended alongside other biologics such as TNFi and IL‐17 inhibitors after DMARD therapy in patients with active PsA.^9^, ^10^ The recommended dose of ustekinumab is 45 mg or 90 mg (subcutaneous) at 0 and 4 weeks and every 12 weeks thereafter.^37^

##### IL‐17 inhibitors

The recommended dose of IL‐17i ixekizumab in patients with active PsA and inadequate response to TNFi agents is starting dose of 160 mg followed by 80 mg every 2 or 4 weeks for the safe and effective management of PsA.^38^ IL‐17 inhibitor secukinumab is administered subcutaneously at doses of 300 mg in patients with concomitant moderate‐to‐severe plaque psoriasis or who are TNFi inadequate responders at weeks 0, 1, 2, 3 and 4 and every 4 weeks thereafter. The loading dose can be 150 mg in other patients at weeks 0, 1, 3, 4 and every 4 weeks thereafter and based on clinical response the dose can be increased to 300 mg.^39^

##### IL‐23 inhibitors

Guselkumab, a specific IL‐23 inhibitor, has been recently approved for the treatment of PsA by the US FDA and European Medicines Agency (EMA).^17^ The recommended dose of guselkumab is 100 mg at weeks 0 and 4, and every 8 weeks thereafter.^40^ Risankizumab was not approved for PsA by the FDA and EMA when our consensus statements were drafted. However, the EMA and FDA approved it on November 22, 2021 and January 21, 2022 respectively for treatment of active psoriatic arthritis.^41^, ^42^

##### CTLA4‐Ig

Abatacept is a biologic agent that targets T‐cell costimulatory signals selectively and is approved for the treatment of PsA patients with inadequate response to csDMARDs, excluding those with uncontrolled skin lesions and axial disease.^43^

#### 3.2.6 Biosimilars

In recent years, biosimilars of infliximab, etanercept, and adalimumab have been approved by regulatory bodies in Europe and the USA for the treatment of PsA. These agents have been approved for the treatment of PsA based on similar efficacy to the reference product in psoriasis and/or rheumatoid arthritis (RA), by the extrapolation principle.^44^, ^45^, ^46^ Biosimilars are more cost‐effective compared to biologics, and thereby represent a solution for better patient accessibility to therapy and reduction in associated healthcare costs.^47^

Consensus statements on dosage recommendations for non‐biologic and biologic therapies are provided in Table 1.

### 3.3 Treatment recommendations based on PsA domains

#### 3.3.1 Peripheral arthritis

In DMARD‐naïve patients with peripheral arthritis, csDMARDs (methotrexate, sulfasalazine, and leflunomide), PDE‐4i, IL‐12/23i, IL‐17i, IL‐23i, JAKi, and TNFi are recommended therapeutic options.^9^, ^11^, ^33^ In patients with monoarthritis or oligoarthritis accompanied by factors such as dactylitis or joint damage, the use of csDMARDs and intra‐articular (IA) GCC should be considered.^9^ In patients with polyarticular disease, csDMARDs should be considered either as first‐line treatment or after a short course of NSAIDs. In patients with inadequate response to csDMARDs, TNFi, IL‐12/23i, IL23i, IL17i, PDE‐4i and JAKi are recommended therapeutic options.^9^, ^10^, ^34^, ^48^, ^49^ In patients with inadequate response to 1 biologic treatment, switching to another biologic within the same drug class, or to a drug with a different mode of action, should be considered.^9^, ^10^ In all patients with peripheral arthritis, IA and systemic GCC are conditionally recommended at the lowest dosages and for a short duration.

#### 3.3.2 Axial disease

At the time of drafting consensus statements, there were no studies on management of psoriatic spondylitis. Therefore, management of this condition depends on current treatment modalities in ankylosing spondylitis. In patients with psoriatic spondylitis/axial disease who are biologic‐naïve, NSAIDs, physiotherapy, simple analgesia, TNFi or IL‐17i, JAKi are recommended therapeutic options. Tofacitinib was not approved for PsA by the FDA and EMA when the consensus statements were drafted. However, FDA and EMA approved it on November 18, 2021 and December 14, 2021 respectively for treatment of ankylosing spondylitis. Other therapeutic options such as sacroiliac joint GCC injections and bisphosphonates can be used, but with caution.^9^, ^14^, ^50^, ^51^, ^52^, ^53^, ^54^, ^55^, ^56^

#### 3.3.3 Enthesitis

For the management of PsA patients with enthesitis (inflammation at the sites of attachment of ligaments, tendons, and joint capsules to bone),^57^ treatment with TNFi, IL‐17i, IL‐23i, IL‐12/23i and JAKi are recommended therapeutic options. Other therapeutic options include NSAIDs, physiotherapy, methotrexate, CTLA4‐Ig, and PDE‐4i.^9^, ^10^, ^49^, ^58^, ^59^, ^60^, ^61^, ^62^

#### 3.3.4 Dactylitis

The recommended therapies for the management of PsA patients with dactylitis include TNFi therapies (infliximab, adalimumab, golimumab, and certolizumab pegol), IL‐17i, IL‐12/23i, IL‐23i, JAKi, and PDE‐4i.^9^, ^58^, ^62^, ^63^ Other therapeutic options include NSAIDs, GCC injections, and methotrexate.^9^

#### 3.3.5 Nail disease

For the management of PsA patients with moderate‐to‐severe nail disease, TNFi, IL‐17i, PDE‐4i, IL‐23i, IL‐12/23i and acitretin are recommended therapeutic options.^9^, ^10^, ^64^, ^65^, ^66^, ^67^ Other options include topical therapies, procedural therapies, csDMARDs (cyclosporine, leflunomide, and methotrexate).^9^, ^68^, ^69^

#### 3.3.6 Skin disease

The expert panel recommends the use of topical therapies, phototherapy, acitretin and csDMARDs (methotrexate, leflunomide, cyclosporine) as first‐line therapeutic options, especially for PsA patients with milder skin disease.^9^ TNFi, IL‐17i, IL12/23i, IL‐23i, JAKi and PDE‐4i are recommended therapeutic options for treatment of PsA patients with significant skin involvement.^9^ Furthermore, biologic agents such as IL‐17i are preferred to TNFi, with or without topical treatments and DMARDs, in PsA patients with active psoriasis (≥3% of body surface area of skin involvement).^70^ In accordance with GRAPPA 2015 recommendations, the expert panel recommends switching from one DMARD to another, or to biologic treatment, or from one biologic treatment to another.^9^

Consensus recommendations for the treatment of PsA domains were based on GRAPPA 2015, GRAPPA 2020, EULAR 2019 recommendations and literature review, and are presented in Table 2.

**TABLE 2:** Consensus recommendations for treatment of PsA domains

Domain	Recommended therapeutic options (strongly recommended)	Other therapeutic options (conditionally recommended)	To be avoided (strongly not recommended)
Peripheral arthritis DMARD‐naïve^9^, ^11^, ^33^	csDMARD TNFi PDE‐4i IL‐12/23i IL‐17i IL‐23i JAKi	NSAIDs GCCs (oral or IA) CTLA‐4 Ig	
Peripheral arthritis DMARD inadequate response^9^, ^10^, ^34^, ^48^, ^49^	TNFi PDE‐4i IL‐12/23i IL‐17i IL‐23i JAKi	NSAIDs GCCs (oral or IA) csDMARD CTLA‐4 Ig	
Peripheral arthritis, inadequate response to biological treatment^9^, ^54^	TNFi IL‐17i IL‐12/23i IL‐23i JAKi	NSAID GCC (oral and IA) PDE‐4i CTLA‐4 Ig	
Axial PsA^9^, ^50^, ^51^, ^52^	NSAIDs and simple analgesia Physiotherapy TNFi IL‐17i JAKi	GCC injection for sacroiliac joints Bisphosphonate	csDMARD IL‐12/23i
Enthesitis^9^, ^10^, ^49^, ^58^, ^59^, ^60^, ^61^, ^62^	TNFi IL‐17i JAKi IL‐23i IL‐12/23i	NSAIDs physiotherapy MTX CTLA‐4 Ig PDE‐4i	
Dactylitis^9^, ^54^, ^58^, ^62^, ^63^	TNFi IL‐17i IL12/23i IL‐23i JAKi PDE‐4i	NSAIDs GCCs injections MTX CTLA‐4 Ig	
Nail disease^9^, ^10^, ^64^, ^65^, ^66^, ^68^, ^69^	TNFi IL‐17i PDE‐4i IL‐12/23i IL‐23i		
Skin disease (plaque)^9^, ^119^, ^120^, ^121^, ^122^, ^123^	Topical therapies, phototherapy Acitretin csDMARDs (MTX, LEF, CSA) TNFi IL‐12/23i IL‐17i IL‐23i JAKi PDE‐4i		

Abbreviations: CSA, cyclosporine; CTLA‐4 Ig, cytotoxic T lymphocyte‐associated antigen‐4 immunoglobulin; DMARD, disease‐modifying antirheumatic drug; GCC, glucocorticoids; IA, intra‐articular; IL‐12/23i, interleukin‐12/23 inhibitor; IL‐17i, interleukin‐17 inhibitor; IL‐23i, interleukin‐23 inhibitor; IV, intravenous; JAKi, Janus kinase inhibitor; LEF, leflunomide; MTX, methotrexate; NSAIDs, non‐steroidal anti‐inflammatory drugs; PsA, psoriatic arthritis; PDE‐4i phosphodiesterase‐4 inhibitor (apremilast); SC, subcutaneous; TNFi, tumor necrosis factor inhibitor.

### 3.4 Treatment response

Evaluating response to therapy in patients with PsA can be difficult due to its complex nature, which encompasses a multitude of clinical manifestations. To date, there is no standardized outcome measure for PsA.

In terms of efficacy and safety profile, experts agreed to adhere to the recommendations from guidelines, latest literature evidence and prescription label. Accordingly, consensus statements were developed for efficacy and safety of non‐biologic and biologic therapies.

### 3.5 Efficacy and safety profile of non‐biologic pharmacological therapies

Consensus statements on efficacy/safety profile of non‐biologic pharmacological therapies are detailed in Table 3

**TABLE 3:** Consensus statements on efficacy/safety profile of non‐biologic pharmacological therapies

*Symptomatic treatments*
NSAIDs In PsA patients with peripheral arthritis, NSAID monotherapy without DMARDs should not exceed 1 mo if disease activity persists.^10^ In the case of axial or entheseal involvement, NSAID therapy may be continued for up to 12 wks if relief has already been achieved after 4 wks.^10^ Because of the potential for side effects (eg gastrointestinal complications, hepatic complications, allergic complications, cardiovascular complications and chronic kidney disease), NSAIDs should be used with caution. NSAIDs such as celecoxib are contraindicated in patients with hypersensitivity to celecoxib, patients with history of asthma, urticaria, or other allergic type of reactions after taking NSAIDs. NSAID use should be avoided during the perioperative period in the setting of coronary artery bypass surgery.
GCCs Systemic GCCs: may be associated with skin flares. Should be used with caution, especially when treatment is being tapered for the potential worsening of skin symptoms. Intra‐articular injection of GCCs may rarely result in depigmentation.^124^
*csDMARDs*
Methotrexate Should be prescribed at an optimal dose of 25 mg per wk and with folate supplementation; if improvement does not exceed 50% of a composite measure for PsA within 3 mo or the treatment target is not reached within 6 mo, JAKi or bDMARD can be added to MTX treatment. Common adverse effects: gastrointestinal manifestations, hepatotoxicity, dizziness, photosensitivity. Should be used with caution in patients with impaired renal function, ascites pleural effusion and avoided in pregnant women due to its teratogenic effects. Other contraindications: liver disease, immunodeficiency syndrome, pre‐existing blood dyscrasias and in patients with hypersensitivity to MTX.
Leflunomide Effective and safe in the management of PsA, particularly in reducing tenderness, pain, fatigue, dactylitis, and skin disease in patients with PsA.^125^ Common adverse effects: diarrhea, nausea, headache, rash, respiratory infection, abnormal liver enzymes. Should be used with caution in patients with severe infections. Caution should be taken for its use in pregnant women and in patients with severe hepatic impairment.
Sulfasalazine Shows greater improvement in patients with symmetrical polyarticular peripheral arthritis. Shows significant improvement in joint scores and reduction in disease activity as early as the 4th wk of treatment.^126^ Well tolerated and safe in patients with PsA at a dose of 2.0 g/d. Patients with PsA who are known or suspected to have COVID‐19, should continue using sulfasalazine.^127^, ^128^ Common adverse events: gastric upset, skin rashes, headache, and liver disorders. Should be used with caution in patients with severe allergy, bronchial asthma, and glucose‐6‐phosphate dehydrogenase deficiency (G6PD). Should be avoided in patients with intestinal or urinary obstruction, porphyria, and hypersensitivity to sulfasalazine.
*tsDMARDs*
Apremilast Effective in the treatment of biologic‐naïve patients with PsA and has a tolerable safety profile. Most common adverse effects: diarrhea and nausea. Should be used with caution in PsA patients with depression. Does not require routine therapeutic monitoring. Safe and effective therapeutic option in the HIV‐infected population with psoriatic arthritis.
Tofacitinib Safe and effective in the management of csDMARD‐IR/ TNFi‐naïve and TNFi‐IR patients and is effective in PsA patients with enthesitis and dactylitis. Patients with recurrent deep‐vein thrombosis and those at high risk of shingles infection should exercise caution. Has an acceptable safety profile with a low incidence of serious infections, malignancies, cardiovascular events, and gastrointestinal complications.
Upadacitinib Safe and effective in the management of patients with active PsA. Common adverse effects: upper respiratory tract infections, nausea, cough, and pyrexia. Patients with active and serious infections, malignancy, thrombosis, and gastrointestinal perforation should be treated with caution.

Abbreviations: bDMARD, biologic disease‐modifying antirheumatic drug; csDMARD, conventional synthetic disease‐modifying antirheumatic drug; COVID‐19, coronavirus disease‐2019; GCC, glucocorticoids; HIV, human immunodeficiency virus; IR, inadequate response; JAKi, Janus kinase inhibitor; MTX, methotrexate; NSAIDs, non‐steroidal anti‐inflammatory drugs; PsA, psoriatic arthritis; TNF, tumor necrosis factor; tsDMARD, targeted synthetic disease‐modifying antirheumatic drug.

### 3.6 Efficacy and safety profile of biologic pharmacological therapies

Consensus statements on the efficacy and safety of biologic pharmacological therapy are presented in Table 4.

**TABLE 4:** Consensus statements on efficacy/safety profile of biologic pharmacological therapy

*TNFi*
Adalimumab at a dose of 40 mg (at baseline, wks 2 and 4, and every 4 wks thereafter), can significantly improve joint and skin manifestations and reduce radiographic progression in patients with active PsA, by achieving clinical response by 6 mo.^129^, ^130^ Etanercept (25 mg twice weekly) can significantly reduce the signs and symptoms of PsA and achieve clinical response by wk 12, with efficacy lasting from 48 wks to 2 y.^108^, ^109^ Infliximab, at a dose of 5 mg/kg (at wks 0, 2, and 6, and every 8 wks thereafter), significantly inhibits the progression of radiographic damage in patients with active PsA as early as 6 mo after starting treatment, and the beneficial effect continues through 1 y of treatment.^131^ Infliximab at a dose of 5 mg/kg significantly improves the signs and symptoms of arthritis, psoriasis, dactylitis, and enthesitis in patients with active PsA resistant to DMARD therapy.^132^, ^133^ Certolizumab pegol (200 mg every 2 wks or 400 mg every 4 wks) provides rapid improvement in the signs and symptoms of PsA, including joints, skin, enthesitis, dactylitis, and nail disease. It can reduce the progression of structural damage for up to 2 y in PsA patients with/without prior TNFi exposure.^134^, ^135^ Golimumab (100 mg) also inhibits radiographic progression as early as 6 mo and is effective in maintaining clinical improvement for 5 y.^136^
TNFi therapy should not be initiated or continued in the presence of serious active infection, but it can be restarted once the infection has clinically resolved. TNFi therapy should be used with caution in patients at high infection risk: o Active mycobacterial infection should be adequately treated before TNFi therapy is started. o HIV or HCV infection should not preclude treatment with TNFi therapy, although treatment should only be commenced in those with well‐controlled disease and with appropriate monitoring under the care of a hepatologist or HIV specialist. Of note, etanercept has been shown to be safe in PsA patients with HCV. o TNFi therapy in those with chronic HBV should be approached with caution, given the potential risk of reactivation and fulminant hepatitis. TNFi therapy should be avoided in patients with a current or prior history of malignancy. Caution should be exercised in patients with serious infections and demyelinating diseases or systemic lupus erythematosus.
*IL‐12/23i*
Ustekinumab (SC) at a dose of 45 mg or 90 mg (weight‐dependent) reduces the signs and symptoms of articular and dermatological manifestations in PsA patients with and without TNFi therapy exposure and is well tolerated through 16 wks of therapy.^137^ Adverse effects of IL‐12/23i: upper respiratory tract infections, nasopharyngitis, back pain, headache, injection‐site reactions, myalgia, fatigue, and rarely severe infection and malignancy. Caution should be taken in patients with serious infections and malignancy. Ustekinumab is contraindicated in patients with clinically significant hypersensitivity to ustekinumab or to any of its excipients.
*IL‐17i*
Secukinumab (SC) improves signs and symptoms in multiple clinical domains in patients with active PsA and is well tolerated through 5 y of therapy.^138^ Secukinumab (150–300 mg) is well tolerated for long‐term treatment. Ixekizumab is a highly effective treatment for active PsA patients, especially those previously exposed to csDMARDs and TNFi therapies.^38^, ^139^ Most common adverse effects associated IL‐17i: upper respiratory tract infections and injection‐site reactions. IL‐17i should be used with caution in patients with concomitant IBD and severe infections. IL‐17i are contraindicated in patients with serious allergic reactions to the molecule or its recipients.
*IL‐23i*
Guselkumab improves joint symptoms significantly, with more than one‐third of patients achieving ACR50 by wk 24.^140^ Guselkumab shows significant improvement in inhibition of radiographic progression of joint structural damage and resolving enthesitis, dactylitis in patients with active PsA at 24 wks.^140^, ^141^ Common adverse events of guselkumab: hypersensitivity reactions, including anaphylaxis and upper respiratory tract infections, gastroenteritis, tinea infections, and herpes simplex infections.
*CTLA4‐Ig*
Abatacept can be effective in patients with PsA refractory to DMARDs.^49^ Most common adverse effects associated with abatacept include headache, upper respiratory tract infection, nasopharyngitis, and nausea.

Abbreviations: ACR, American College of Rheumatology; CTLA4‐Ig, cytotoxic T‐lymphocyte‐associated protein 4‐immunoglobulin; DMARD, disease‐modifying antirheumatic drug; HBV, hepatitis B virus; HCV, hepatitis C virus; HIV, human immunodeficiency virus; IBD, inflammatory bowel disease; IL‐12/23i, interleukin‐12/23 inhibitor; IL‐17i, interleukin‐17 inhibitor; IL‐23i, interleukin‐23 inhibitor; PsA, psoriatic arthritis; SC, subcutaneous; TNFi, tumor necrosis factor inhibitor.

### 3.7 Treatment failure and switching therapy

The expert panel agreed that transitioning to alternative TNFi should be considered in PsA patients with primary or secondary failure of TNFi therapy. The response to alternative treatment should be assessed with the same criteria as those used for the first TNFi agent. Furthermore, possible consequences for control of skin disease should be considered and referral to a dermatologist also considered, if required.^11^

Similarly, switching within a class or between a class (bDMARD to tsDMARD) can also be considered in cases of primary or secondary failure of a bDMARD. However, it is more advisable to change class after a second failure within a given class.^11^

### 3.8 Screening and monitoring requirements for PsA therapies

The expert panel suggested that before initiation of any systemic therapy, a practical approach should be adopted to monitor PsA patients based on medical history, physical examination, and tests (laboratory and imaging). The expert panel opined that monitoring of systemic therapies is crucial to maximize the benefits and minimize the risks associated with these drugs.

Owing to an increased risk of hepatotoxicity and renal toxicity associated with most systemic DMARDs, experts recommended monitoring tests including complete blood count, comprehensive liver function tests, renal function test, erythrocyte sedimentation rate (ESR), C‐reactive protein (CRP), and serum creatinine levels.

As most bDMARDs are immunomodulators, there is a high risk of serious infections, including tuberculosis, hepatitis, and human immunodeficiency virus (HIV). Therefore, it is important that patients are routinely screened for tuberculosis (QuantiFERON), hepatitis B virus (HBV), hepatitis C virus (HCV), and HIV prior to initiating any biologic therapy.

Furthermore, patients should be assessed for their vaccination status before initiating any systemic therapy. Routine vaccination for pertussis and inactivated influenza, pneumococcal, and HBV is recommended in high‐risk patients and in highly prevalent regions at baseline. Varicella‐zoster antibody (IgG) test, especially in patients taking JAKi, should be conducted at baseline. Live vaccines should be avoided during treatment with biologics.

In accordance with the recommendations provided by the Saudi guideline and GRAPPA 2015 and evidence from the literature, the expert panel agreed upon consensus statements on screening and monitoring requirements for PsA therapies presented in Table 5.

**TABLE 5:** Consensus statements on screening and monitoring requirements for PsA therapies

*Recommendations*
Recommended laboratory tests prior to initiation of a biologic treatment include:^9^, ^13^ o Complete blood count: hemoglobin, hematocrit, white blood cell count, white blood cell differentiation, and platelet count o Comprehensive liver function tests: direct bilirubin, total bilirubin, ALP, ALT, and GGT o Renal function test Other important tests include ESR, CRP, and serum creatinine.
Screening for HIV, HBV, HCV, and tuberculosis (QuantiFERON preferable) should be strongly considered, in accordance with local guidelines and standards of medical practice before initiation of therapies that may potentially alter normal immune response.^9^
Inclusion of varicella‐zoster antibody (IgG) test, especially in patients taking JAKi, for baseline tests, should be considered.^142^
Chest X‐ray as a baseline monitoring test for all drugs should be strongly considered.
A general screening questionnaire for malignancy should be considered on a case‐by‐case basis.
Given the increased prevalence and incidence of CVD and diabetes among patients with PsA, regular screening is recommended (eg Framingham risk score or ASCVD for CVD risk assessment).^9^
Screening for depression and anxiety among patients with PsA should also be considered.^9^
Given the association of ophthalmic disease with spondylarthritis and an increased risk of IBD among patients with PsA, consideration of screening for eye disease and Gastrointestinal disease is recommended as a part of the review of systems, as well as consideration of appropriate referral, as applicable.^9^
Monitoring tests should be conducted every 1–3 mo during treatment and based on clinical judgment.

Abbreviations: ALP, alkaline phosphatase; ALT, alanine transaminase; ASCVD, atherosclerotic cardiovascular disease; CRP, C‐reactive protein; CVD, cardiovascular disease; ESR, erythrocyte sedimentation rate; GGT, G‐glutamyl transferase; HBV, hepatitis B virus; HCV, hepatitis C virus; HIV, human immunodeficiency virus; IBD, inflammatory bowel disease; IgG, immunoglobulin G; JAKi, Janus kinase inhibitor; PsA, psoriatic arthritis.

### 3.9 Management of comorbidities

Identifying comorbidities is critical to the optimal management and treatment of PsA. Common comorbidities in patients with PsA include CVD, obesity, metabolic syndrome, hypercholesterolemia, hypertension, diabetes mellitus, chronic kidney disease, malignancy, osteoporosis, non‐alcoholic fatty liver disease (NAFLD), and depression. Moreover, some comorbidities such as inflammatory bowel disease (IBD) and ophthalmic disease (eg uveitis) might present as extra‐articular manifestations of disease.

#### 3.9.1 CVD

The risk of major adverse cardiovascular events has been found to be higher in patients with PsA not prescribed DMARDs compared to the general population or those with psoriasis only.^71^, ^72^ Based on clinical evidence and GRAPPA 2020 recommendations, JAKi, ustekinumab, IL‐17i, IL‐23i, abatacept could be considered as treatment options in management of PsA patients with comorbid CVD.^14^, ^54^, ^73^, ^74^, ^75^, ^76^ GRAPPA recommends caution with the use of TNFi, glucocorticoids and NSAIDs in patients with congestive heart failure (CHF).^9^

#### 3.9.2 Obesity and metabolic syndrome

GRAPPA 2020 recommends that physicians should be cautious about prescribing glucocorticoids to patients with metabolic syndrome, and methotrexate to patients with obesity and metabolic syndrome.^14^, ^54^

#### 3.9.3 Hypercholesterolemia

He expert panel emphasized the importance of lipid‐lowering drugs and nutritionist referral in PsA patients with comorbid hypercholesterolemia. Evidence suggests that tofacitinib should be used with caution in PsA patients with comorbid hypercholesterolemia.^77^

#### 3.9.4 Hypertension

Statins, angiotensin‐converting enzyme inhibitors, and/or angiotensin II blockers are preferred treatment options in patients with PsA and hypertension.^9^ Caution should be taken when prescribing NSAIDs, cyclo‐oxygenase‐2 (COX2) inhibitors, or prednisone, as they are associated with an increased risk of CVD.^9^, ^78^

#### 3.9.5 Diabetes mellitus

The prevalence of type 2 diabetes mellitus in patients with PsA has been reported to be between 6.1% and 20.2% with a higher risk noted in women with more severe forms of PsA.^79^ When selecting the treatment for PsA in such patients, most guidelines recommend taking caution with glucocorticoids and methotrexate, as they could worsen glycemic homeostasis and/or influence cardiovascular risk factors such as arterial hypertension.^9^, ^11^

#### 3.9.6 IBD

There is increased prevalence of IBD and subclinical bowel inflammation among patients with PsA.^80^, ^81^ Sulfasalazine, TNFi, and tofacitinib (only ulcerative colitis) are approved treatments for IBD. According to 2018 ACR/NPF guidelines, for patients with active PsA and concomitant active IBD who are DMARD‐naïve, monoclonal TNFi are the preferred choice.^11^ In patients who are contraindicated for TNFi, IL‐12/23i can be prescribed. The use of NSAIDs and IL‐17i should be avoided, as they may exacerbate IBD symptoms.^82^, ^83^ Interim analysis reports from a phase II study suggest that guselkumab could be effectively used in patients with Crohn's disease.^84^ A long‐term study on the efficacy of adalimumab in the treatment of patients with Crohn's disease with intolerance or inadequate response to infliximab reported sustained clinical remission and response with adalimumab maintenance therapy.^85^

#### 3.9.7 Uveitis

The management of concomitant uveitis in PsA patients varies depending on the severity of the disease and its impact on daily activities.^86^ Both infliximab and adalimumab are effective treatment options^87^, ^88^, ^89^ certolizumab pegol/golimumab has shown moderate success, while etanercept has demonstrated only limited success.^90^ Secukinumab showed promising results in phase II clinical trials; however, primary efficacy endpoint was not met in the phase III study^91^, ^92^ The efficacy of IL‐17i, IL‐12/23i, and JAK/STAT (signal transducer and activator of transcription) inhibitors is currently under evaluation.^91^, ^92^

#### 3.9.8 Depression

The prevalence of depression and anxiety among patients with PsA is 9% to 36% and 15% to 30%, respectively.^93^, ^94^ As both depression and anxiety may affect pain perception, QoL, and treatment outcomes; it is important to take appropriate screening measures and treatment decisions in such patients. Apremilast should be used with caution in patients with PsA and comorbid depression.^95^

#### 3.9.9 Hyperuricemia and gout

Hyperuricemia is common in patients with PsA, especially in those with longer CVD, metabolic syndrome, and disease duration.^96^, ^97^, ^98^ It is therefore important to regularly monitor serum uric acid levels in patients with PsA.^97^ Gout is an important differential diagnosis of PsA and, therefore, awareness about its increased incidence in this population is critical.^99^

#### 3.9.10 Hypothyroidism

Due to increased incident cases of hypothyroidism, thyroid dysfunction, positive antithyroid peroxidase antibodies (AbTPO), and appearance of a hypoechoic thyroid pattern in patients with PsA, especially women, it is important to evaluate AbTPO levels, thyroid function, and thyroid ultrasound, with regular follow‐up visits.^100^, ^101^

#### 3.9.11 Osteoporosis

Screening for osteoporosis in psoriatic patients is performed by measuring bone mineral density through dual‐energy X‐ray absorptiometry (DEXA) or assessing the Fracture Risk Assessment (FRAX) score. For management, specific guidelines should be followed for patients treated with chronic systemic glucocorticoids, as it may modify bone mineral density due to bone loss.^102^

#### 3.9.12 Malignancy

Given the potential risk of de novo or recurrent malignancy being associated with the use of TNFi, regular screening is recommended, especially in patients with a history of cancer.^9^ In contrast, IL‐17i, abatacept have a better safety profile for malignancy and are preferred treatment options in these patients.

#### 3.9.13 Fatty liver disease

Liver disease, particularly NAFLD, has an increased prevalence in patients with psoriasis and PsA.^103^ Given the potential risk of liver damage with specific PsA treatments, regular monitoring of liver function abnormalities is deemed necessary. Liver biopsy should also be considered, based on the presence or absence of risk factors for hepatotoxicity and cumulative methotrexate dose.^9^, ^11^ Furthermore, caution should be taken when prescribing methotrexate, leflunomide, sulfasalazine, and NSAIDs in patients with established liver disease due to the increased risk of hepatotoxicity.

#### 3.9.14 Chronic kidney disease

Methotrexate should be avoided in patients with significant renal insufficiency or end‐stage renal disease on hemodialysis, given that renal impairment is a major risk factor for developing methotrexate toxicity.^104^ NSAIDs should also be avoided, given that they may increase the risk for acute kidney injury.^105^

#### 3.9.15 Serious infections

There is a high risk of serious infections, including tuberculosis, hepatitis, and HIV, associated with the use of certain PsA treatments—including TNFi.^9^ Due to the low incidence of serious infections observed with IL‐12/23i, IL‐17i, and abatacept in comparison to TNFi, the former are preferred treatment options for PsA.^9^

Consensus statements on the management of comorbidities in patients with PsA are presented in Table 6.

**TABLE 6:** Consensus statements on management of comorbidities

Comorbid condition/s	Treatment options/referral/monitoring	Treatment requiring caution
Cardiovascular disease and congestive heart failure	JAKi, ustekinumab, IL‐17i, IL‐23i, abatacept^73^, ^74^, ^75^, ^76^	NSAIDs, GCCs, TNFi (TNFi should be avoided in patients with severe CHF [NYHA class III and IV] and should be used with caution in patients with mild CHF [NYHA class I and II])^9^, ^54^, ^143^, ^144^
Obesity and metabolic syndrome	Weight reduction, nutritionist referral, obesity/endocrine clinic referral	MTX, GCCs^9^
Hypercholesterolemia	Lipid‐lowering agents, nutritionist referral	Tofacitinib^77^
Hypertension	Statins, angiotensin‐converting enzyme inhibitors, and/or angiotensin II blockers^9^	NSAIDs, GCCs^9^
Diabetes mellitus	Hypoglycemic medications	MTX, GCCs^9^
IBD	TNFi (excluding etanercept) for UC (tofacitinib, IL‐12/23i) IL‐23i^84^	IL‐17i
Uveitis	TNFi (especially adalimumab and infliximab), MTX	NSAIDs, IL‐17i^145^
Depression	Psychiatry referral	Apremilast
Hyperuricemia and gout	Monitoring serum uric acid levels Urate‐lowering therapy if indicated	
Thyroid disease	Routine thyroid tests/endocrine referral	
Osteoporosis	Monitor with DEXA as indicated in non‐PsA patients	GCCs
Malignancy	Oncology referral, IL‐17i, abatacept	All biological agents
Fatty liver disease	GI referral Weight loss Dietician referral	NSAIDs, SSZ, MTX, LEF, tofacitinib^54^
Chronic kidney disease	Nephrologist referral	NSAIDs, MTX^54^
HBV	Ustekinumab GI referral, monitor HBV PCR (once a mo for first 3 mo and every 3 mo thereafter)^146^	NSAIDs, MTX, LEF, biologics (for carriers)^54^
HCV	GI referral, monitor HCV PCR (once in every 3‐6 mo)^146^	NSAIDs, MTX, LEF, biologics (for carriers)^54^
Tuberculosis	IL‐17i, abatacept, referral to respiratory or infectious disease specialist	TNFi especially infliximab

Abbreviations: CHF, congestive heart failure; DEXA, dual‐energy X‐ray absorptiometry; GCCs, glucocorticoids; GI, gastrointestinal; HBV, hepatitis B virus; HCV, hepatitis C virus; IBD, inflammatory bowel disease; IL‐12/23i, interleukin‐12/23 inhibitor; IL‐17i, interleukin‐17 inhibitor; IL‐23i, interleukin‐23 inhibitor; JAKi, Janus kinase inhibitor; LEF, leflunomide; MTX, methotrexate; NHYA, New York Heart Association; NSAIDs, non‐steroidal anti‐inflammatory drugs; PCR, polymerase chain reaction; SSZ, sulfasalazine; TNFi, tumor necrosis factor inhibitor; UC, ulcerative colitis.

#### 3.9.16 Immunization

Immunization status of the patient should be assessed. Routine vaccination for pertussis and inactivated influenza, pneumococcal, and HBV (in high‐risk patients and in highly prevalent regions) should be performed at baseline.^9^

## 4 CONCLUSION

The present consensus statements for the pharmacological management of PsA are in corroboration with established global guidelines on the different aspects of PsA, especially highlighting the management of PsA and associated comorbid conditions and monitoring of therapies in patients with PsA. There is a scarcity of such consensus‐based statements in the Arab world. Furthermore, our consensus statements are aligned with the most recently published Saudi consensus recommendations.^106^ Our detailed consensus recommendations can help physicians and healthcare professionals in the UAE to make informed treatment decisions, improvise treatment strategies, monitor therapies, as well as effectively manage comorbidities in patients with PsA.

## AUTHOR CONTRIBUTIONS

KAA had a substantive role in drafting the final manuscript. The authors are fully responsible for all the content and editorial decisions; the authors involved themselves at all stages of manuscript development and approved the final version.

## CONFLICT OF INTEREST

The authors have no conflict of interest related to this work. The consensus guideline was funded by the Emirates Society for Rheumatology, a non‐profit organization.
